# Complete Genome Sequences of Two Environmental *Legionella* Isolates Obtained from Potable Water Sourced in a First Nation Community

**DOI:** 10.1128/MRA.01237-20

**Published:** 2021-01-28

**Authors:** Teassa L. MacMartin, Christopher I. Graham, Annemieke Farenhorst, Ann Karen C. Brassinga

**Affiliations:** a Department of Microbiology, Faculty of Science, University of Manitoba, Winnipeg, Manitoba, Canada; b Department of Soil Science, Faculty of Agricultural and Food Sciences, University of Manitoba, Winnipeg, Manitoba, Canada; University of Maryland School of Medicine

## Abstract

Here, we report the complete genome sequences of two distinct isolates of *Legionella* that were obtained from potable water sourced from cistern-bearing homes within a First Nation community in Manitoba, Canada.

## ANNOUNCEMENT

*Legionella* is a Gram-negative genus of bacteria that inhabits freshwater and soil, persisting either in planktonic form or as an intracellular parasite of bacterivorous protozoan hosts (1). It can colonize biofilms in drinking water distribution infrastructure (2), necessitating frequent monitoring of water quality to reduce the risk of the respiratory infection Legionnaires’ disease, particularly for immunocompromised individuals (3). Due to the frequency of drinking water advisories (4) as well as recent studies highlighting the presence of fecal bacteria in potable water of First Nation communities in Canada (5, 6), we sought to assess the presence and potential health risks of *Legionella* species. In 2018, a pilot study was initiated in collaboration with a First Nation community (Manitoba, Canada) to sample homes piped directly by a water treatment plant (WTP) or using cisterns supplied by WTP transport trucks.

*Legionella* was cultured from concentrated water samples or biofilm samples collected from shower heads within homes by use of buffered charcoal-yeast extract (BCYE) agar or BCYE agar with glycine-vancomycin-polymyxin-cycloheximide (GVPC) antibiotic supplementation (7) (Oxoid Ltd.), with or without acid (0.2 M KCl/0.2 M HCl [18:1] [∼pH 2.0]; 15-min incubation) (8) or heat (modified from 30 to 45 min at 50°C) (9) pretreatment for enrichment of *Legionella* species. Isolates were then verified by PCR amplification of *ssrA* (for *Legionella* spp.) and *mip* (with specificity for Legionella pneumophila) (10).

Two unique isolates were selected for whole-genome sequencing: (i) PC997, collected from a heat-treated, concentrated water sample, and (ii) PC1000, collected from an acid-treated biofilm sample. Each isolate was grown on BCYE agar at 37°C with 5% CO_2_ for 3 to 4 days, and then genomic DNA was purified by phenol-chloroform extraction. Library preparation and sequencing were outsourced to Génome Québec (Montréal, Québec, Canada). For strain PC997, 7.5 μg high-molecular-weight genomic DNA was sheared (Covaris g-TUBEs, at 4,000 rpm, for 60 s on each side with Eppendorf centrifuge 5424); however, DNA for strain PC1000 (6.0 μg) was not sheared because it appeared fragmented at the time of submission. The SMRTbell template preparation kit v1.0 was used for DNA damage repair, end repair, and adapter ligation according to protocol, and the BluePippin size selection system protocol was utilized with a cutoff range of 15 to 50 kb for sample PC997 and a cutoff range of 9 to 50 kb for sample PC1000. Subsequently, the sequencing primer (0.8333 nM) was annealed and P6 v2 polymerase (0.500 nM) was bound. Single-molecule real-time (SMRT) sequencing was performed on a PacBio RS II instrument (Pacific Biosciences, Menlo Park, CA, USA) using the DNA sequencing reagent kit 4.0 v2, SMRT cells v3, and 4-h movies. Sequencing of PC997 resulted in 105,614 filtered reads with an *N*_50_ of 16.0 kb, and sequencing of PC1000 resulted in 89,878 filtered reads with an *N*_50_ of 13.5 kb. Prior to assembly, a BLAST search was implemented for a subset of sequence reads to detect contamination through the Génome Québec Nanuq server. *De novo* assembly was performed by Hierarchical Genome Assembly Process 4 (HGAP4) with PacBio SMRT Link software v7.0.0.63985 (11), and circularization was performed using Circlator v1.5.5 (12). Assembly of PC997 produced three contigs (30× coverage), including a chromosome (3,750,082 bp, with a GC content of 37.9%) and two plasmids (pPC997_1, 150,431 bp, with a GC content of 38.1%; pPC997_2, 79,080 bp, with a GC content of 35.2%), with a total genome size of 3,979,593 bp. Four contigs were assembled from PC1000 (30× coverage), including a chromosome (4,081,644 bp, with a GC content of 38.2%) and three plasmids (pPC1000_1, 68,633 bp, with a GC content of 39.8%; pPC1000_2, 67,880 bp, with a GC content of 40.9%; pPC1000_3, 31,534 bp, with a GC content of 39.2%), with a total genome size of 4,249,691 bp. Legionella anisa (GenBank accession number GCF_001467525.1) was used by the Canadian Center for Computational Genomics (C3G) to guide Prokka v1.14 annotation (13), as the closest identified species to PC997 and PC1000 by 16S rRNA sequencing was L. anisa (GenBank accession number CP029563.1), with 98.71% and 99.19% similarity, respectively. Default parameters were used for all software. The genome of PC997 contained a total of 3,495 predicted coding sequences (CDSs), including 11 rRNAs and 43 tRNAs, whereas PC1000 contained 3,781 predicted CDSs, including 9 rRNAs and 44 tRNAs. Additionally, full Dot/Icm type IVB secretion system components (excluding *icmR*) and core effectors were identified in both isolates (14, 15).

### Data availability.

The genome sequences have been deposited in GenBank under the following accession numbers: *Legionella* sp. isolate PC997, CP059576 (chromosome), CP059577 (pPC997_1), and CP059578 (pPC997_2); *Legionella* sp. isolate PC1000, CP059400 (chromosome), CP059403 (pPC1000_1), CP059401 (pPC1000_2), and CP059402 (pPC1000_3). Raw reads are available through NCBI BioProject number PRJNA640050.
